# Unveiling Uncommon: Perforated Peptic Ulcer Mimicking Post-Cesarean Complications—A Case Report

**DOI:** 10.3390/reports9010092

**Published:** 2026-03-22

**Authors:** Samantha Davis, Samie A. Rizvi, Moosa Malik, Mostafa E. Nagy, Hani Serag

**Affiliations:** 1 John Sealy School of Medicine (JSSOM), University of Texas Medical Branch at Galveston (UTMB), Galveston, TX 77555, USA; samadavi@utmb.edu (S.D.); sarizvi@utmb.edu (S.A.R.); momalik@utmb.edu (M.M.); 2 Faculty of Medicine, Ain Shams University, Cairo 11591, Egypt; mostafanagy@med.asu.edu.eg; 3 John Sealy School of Medicine (JSSOM) and School of Public and Population Health (SPPH), University of Texas Medical Branch at Galveston (UTMB), Galveston, TX 77555, USA

**Keywords:** perforated peptic ulcer, post-cesarean section complications, global health

## Abstract

**Background and Clinical Significance**: Peptic ulcer perforation is a severe complication of peptic ulcer disease, resulting from erosion of the upper gastrointestinal mucosa. While uncommon in post-cesarean patients, its symptoms can resemble post-operative complications, risking delayed diagnosis and adverse outcomes. This case highlights the need for an expanded diagnostic approach in post-cesarean patients with atypical abdominal symptoms. **Case Presentation**: A 27-year-old West African woman presented to Ain Shams University Hospital in Cairo, Egypt, with worsening abdominal pain, vomiting, fever, and tachycardia three weeks post-cesarean. Initially misdiagnosed with gastroenteritis, she underwent emergency laparotomy due to persistent symptoms, which revealed a 3 cm perforated peptic ulcer. An omental patch repair was performed, and she was discharged in stable condition seven days later. Peptic ulcer perforation, although rare post-cesarean, can mimic common post-operative symptoms, leading to diagnostic delays. A thorough evaluation of abdominal symptoms unresponsive to standard post-operative care is essential, as misdiagnosis increases risks of morbidity. Non-gynecologic causes should be considered, particularly with persistent symptoms. **Conclusions**: Physicians should consider peptic ulcer perforation in post-cesarean patients presenting with sustained abdominal pain, fever, or gastrointestinal distress. Recognizing atypical complications early allows timely intervention, improving outcomes and reducing mortality. This case underscores the value of broad differential diagnoses in post-operative care.

## 1. Introduction and Clinical Significance

Peptic ulcer disease (PUD) results from mucosal injury in the upper gastrointestinal tract, where breakdown of protective defenses allows acid/pepsin-related damage to extend beyond the superficial mucosa and form ulcers—most commonly in the stomach and proximal duodenum [1]. Contemporary reviews continue to identify *Helicobacter pylori* infection and nonsteroidal anti-inflammatory drug (NSAID) exposure as the dominant etiologic drivers of PUD [2,3]. These factors compromise the mucosal barrier through distinct (and sometimes synergistic) mechanisms: *H. pylori* promotes chronic gastritis and inflammatory signaling and can dysregulate gastrin–somatostatin pathways, contributing to increased gastric acid secretion in relevant phenotypes [4]. In contrast, NSAIDs cause injury largely via cyclooxygenase (COX) inhibition (especially COX-1), leading to reduced prostaglandin synthesis and downstream decreases in mucus/bicarbonate secretion, mucosal blood flow, and epithelial repair, thereby weakening mucosal defense and increasing susceptibility to acid-related injury and ulceration [5].

In severe cases, an ulcer can deepen and erode through the gastric or duodenal wall, potentially reaching the serosal layer. If perforation occurs, gastric or duodenal contents may spill into the peritoneal cavity, causing a life-threatening condition known as peritonitis. Perforated peptic ulcers present with sudden, severe abdominal pain, often described as “tearing” or “burning,” along with signs of acute abdomen, such as rigidity and rebound tenderness. If left untreated, this can rapidly lead to sepsis and organ failure [6,7].

The occurrence of perforated peptic ulcers in post-operative cesarean section patients is exceptionally rare, as most post-cesarean complications involve the large bowel or other pelvic organs. Pregnancy itself is thought to offer a protective effect against peptic ulcers. Positive Lifestyle changes during pregnancy such as decreased smoking and alcohol intake, as well as improved diet are associated with lowering the risk of PPU. Additionally, hormonal changes during pregnancy may lead to decreased gastric acid secretion. The physiological increase in mucus production and prostaglandins during pregnancy enhances mucosal protection, reducing the likelihood of ulcer formation and progression [8,9].

Despite this rarity, it is crucial for healthcare providers to consider perforated peptic ulcers in the differential diagnosis when caring for post-operative cesarean section patients presenting with symptoms of peritonitis. Overlap in clinical presentation, such as abdominal pain, tenderness, and guarding can make it challenging to distinguish between post-cesarean complications and a perforated peptic ulcer. Misdiagnosis or delayed diagnosis can lead to increased morbidity. This report presents a case that underscores the importance of maintaining a high index of suspicion for perforated peptic ulcers in post-operative cesarean section patients with suspected peritonitis, enabling timely diagnosis and appropriate intervention [10,11].

## 2. Case Presentation

A 27-year-old West African woman, who delivered a healthy baby via an uncomplicated Cesarean section three weeks prior, presented with worsening abdominal pain, vomiting, fever, and tachycardia. The patient was a migrant passing through Egypt and had limited medical documentation. No prior medical, surgical, or family history could be obtained, and there was no known history or peptic ulcer disease or chronic medication use. The patient also denied alcohol use or tobacco use.

She had initially been managed as an outpatient for presumed gastroenteritis but presented to the hospital approximately 4–5 days later with worsening symptoms and signs of systemic illness. On arrival, she was hypotensive (blood pressure of 80/40 mmHg), tachycardia (heart rate of 140 bpm), and febrile (temperature of 40 °C). Initial clinical suspicion was hypovolemic shock secondary to dehydration from prolonged vomiting and suspected gastroenteritis, and immediate fluid resuscitation was initiated.

Laboratory investigations revealed a white blood cell count of 36,000/µL (*reference range*: 4000–11,000/µL), hemoglobin of 10 g/dL (*reference range*: 12–16 g/dL), platelets of 101,000/µL (*reference range*: 150,000–450,000/µL), an INR of 1.84 (*reference range*: 0.8–1.2), creatinine of 1.4 mg/dL (*reference range*: 0.6–1.1 mg/dL), sodium of 130 mmol/L (*reference range*: 135–145 mmol/L), potassium of 4.1 mmol/L (*reference range*: 3.5–5.0 mmol/L), total indirect bilirubin of 7.7 mg/dL (*reference range*: 0.2–0.8 mg/dL), total direct bilirubin of 5.2 mg/dL (*reference range*: 0.0–0.3 mg/dL), AST of 34 U/L (*reference range*: 10–40 U/L), ALT of 16 U/L (*reference range*: 7–56 U/L), and albumin of 1.4 g/dL (*reference range*: 3.5–5.0 g/dL). Lactate measurements and formal severity scoring systems (SOFA or APACHE II) were not available in the community hospital. Viral serologies were negative.

**Figure 1:**
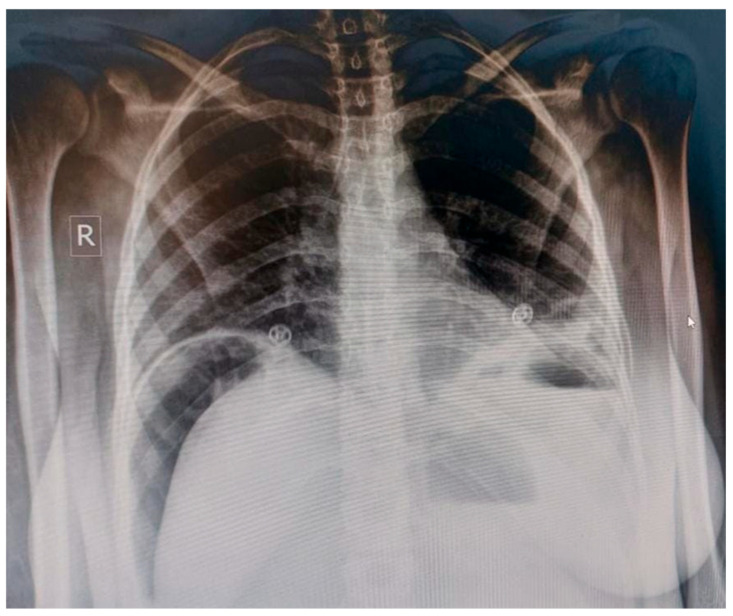
**Abdominal radiograph demonstrating pneumoperitoneum.** Erect abdominal X-ray showing free subdiaphragmatic air (arrow) consistent with perforated viscus, alongside dilated bowel loops and diffuse subcutaneous edema. These findings raised suspicion for intra-abdominal perforation prior to exploratory laparotomy.

She was started on Ceftriaxone and Metronidazole for broad spectrum coverage. An abdominal X-ray (Figure 1) revealed pneumoperitoneum, diffuse subcutaneous edema, and dilated bowel loops with sluggish motility. Given the patient’s clinical instability and the presence of radiographic free air suggestive of perforated viscus, emergent surgical intervention was prioritized. Computed tomography (CT) imaging was not performed, as the combination of clinical findings and diagnostic X-ray findings was considered sufficient to proceed to exploratory laparotomy. Additionally, CT imaging availability was limited in this resource-constrained hospital setting.

**Figure 2:**
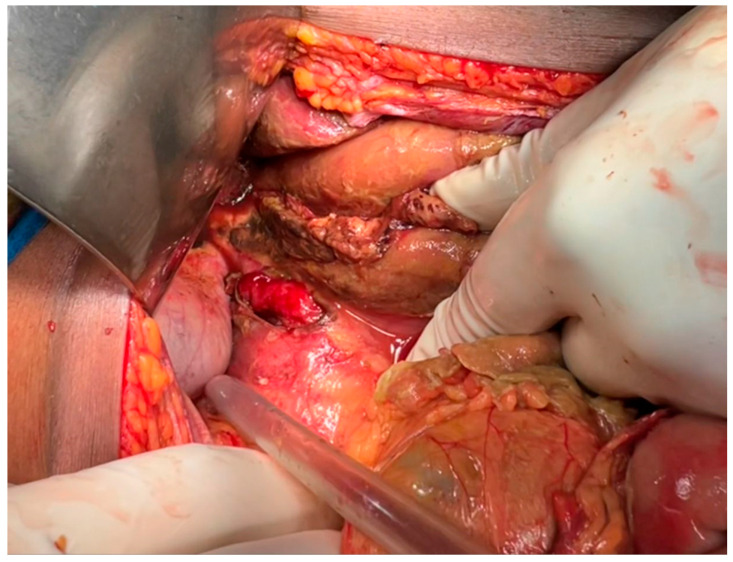
**Intraoperative findings and omental patch repair.** Intraoperative image depicting a 3 cm anterior gastric perforation repaired using an omental (Graham) patch technique. Surrounding peritoneal lavage fluid and surgical drains are visible, illustrating the operative management of the perforated peptic ulcer that was initially misdiagnosed as post-cesarean complications.

The patient underwent an emergency exploratory laparotomy, which identified a 3 cm perforated peptic ulcer. A peritoneal lavage was performed, and the ulcer was repaired using an omental patch (Figure 2). General anesthesia with endotracheal intubation was used. Induction was performed using rapid-sequence induction (RSI) due to risk of aspiration (vomiting, acute abdomen). Typical maintenance was performed with inhalational anesthetic agents and intravenous analgesics and muscle relaxants. Standard ASA (American Society of Anesthesiologists) monitoring (ECG, pulse oximetry, blood pressure, capnography, temperature) was used plus urine output and invasive arterial pressure monitoring due to the patient’s hemodynamic instability. Two surgical drains were placed, and the patient was transferred to the intensive care unit (ICU) for post-operative care.

On the second post-operative day, laboratory results showed normalization of hemoglobin, total direct bilirubin of 3.2 mg/dL, platelets of 122,000/µL, and an INR of 1.6. She tolerated oral fluids by day three, and by day five, she progressed to a soft diet.

Due to the patient’s clinical stabilization and limited diagnostic resources available in treating community hospital, further etiologic testing such as *Helicobacter pylori* assays or endoscopic evaluation was not performed prior to discharge. The patient was discharged in stable condition on the seventh day with recommendations for outpatient follow-up and evaluation. Please refer to the detailed timeline in Table 1.

## 3. Discussion

This case underscores a rare but serious complication in a post-cesarean section patient, perforated peptic ulcer (PPU). While peptic ulcer disease (PUD) is a well-known condition, its progression to perforation in the puerperium is exceptionally uncommon, particularly following cesarean delivery. The overlap in clinical symptoms between typical post-operative complications, such as ileus, bowel injury, and PPU creates significant diagnostic challenges. In our case, the patient’s presentation with abdominal pain, fever, tachycardia, and vomiting was initially attributed to gastroenteritis. This misdiagnosis delayed surgical intervention, increasing the patient’s risk for morbidity. Prompt recognition of PPU is essential, as mortality rates can range from 10% to 28% in developing countries, largely influenced by delays in diagnosis and limited access to surgical care [9,10,12].

Although rare, PPU cases have been previously documented in postpartum women. A systematic review described cases of PPU during pregnancy or the puerperium, emphasizing that while early surgical intervention improved fetal outcomes, maternal outcomes were generally unaffected [11]. Notably, most patients in that review reported a history of dyspepsia or peptic symptoms prior to perforation, information that was not available in our case due to limited documentation. Our patient’s case also differs in its timeline, as most reported cases occurred within 3 to 8 days postpartum, whereas this patient presented 21 days after cesarean delivery. Shirazi et al. similarly noted that delayed recognition of PPU often necessitated re-laparotomy and increased the risk of fatal complications, such as sepsis or septic shock [10]. Despite these parallels, our case stands out due to the prolonged presentation time and absence of known *Helicobacter pylori* infection [10].

The underlying etiology of the perforated peptic ulcer in this patient remains uncertain. Due to the patient’s migrant status and lack of available prior medical records, a comprehensive pre-existing gastrointestinal history could not be established. Additionally, confirmatory testing for *Helicobacter pylori* infection was not available during the acute hospitalization. The patient also denied known NSAID use; however, undocumented exposure to over-the-counter medications or traditional remedies could not be excluded. Other potential contributing factors include physiologic stress associated with recent surgery and the postpartum period, which may predispose patients to stress-related mucosal disease. Postoperative NSAID administration for cesarean pain control is also a recognized risk factor for mucosal injury. In the absence of identifiable classic risk factors, the ulcer in this case may represent multifactorial stress-related mucosal injury occurring in the context of recent surgery, postpartum physiological stress, and potential undocumented medication exposure.

Physiologically, pregnancy is generally considered protective against peptic ulcer formation. This protective effect may be due to increased levels of plasma histaminase, which degrade maternal histamine and consequently reduce gastric acid secretion [13]. Additionally, rising progesterone levels during pregnancy have been associated with decreased gastrointestinal motility, increased mucus secretion, and potential modulation of immune responses in the gastrointestinal tract [14]. These changes, along with common recommendations during pregnancy to avoid ulcerogenic substances like NSAIDs, alcohol, and tobacco, contribute to the reduced incidence of peptic ulcers during gestation. Moreover, elevated epidermal growth factor (EGF) levels and a focus on improved diet and rest during pregnancy may also provide mucosal protection [15]. Despite these physiological safeguards, the development of a perforated ulcer in this patient highlights that PPU, though improbable, remains possible and must not be overlooked.

While abdominal X-rays revealing pneumoperitoneum provided a crucial clue, initial misdiagnosis and delayed imaging contributed to the patient’s clinical deterioration. Various studies have demonstrated the utility of both abdominal X-ray and transabdominal ultrasound in identifying free air and intra-abdominal fluid, respectively [11]. Notably, Shirazi et al. (2020) advocated for the use of bedside ultrasound due to its non-invasiveness, lack of radiation, and cost-effectiveness, advantages particularly relevant for pregnant or recently postpartum patients in resource-limited settings [10]. In our case, laboratory studies also supported the diagnosis by ruling out alternative causes and monitoring post-operative recovery, although they were not diagnostic in isolation.

Once identified, management of PPU involves urgent surgical intervention. The standard procedure, a Graham patch repair using a segment of omentum was performed in this case with good clinical response. The patient stabilized post-operatively, tolerated oral intake by the third day, and was discharged in stable condition by the seventh day. However, this favorable outcome does not negate the fact that earlier recognition and treatment could have reduced the severity of her presentation and the need for ICU-level care. This case emphasizes the need for high clinical suspicion and timely surgical consultation when post-cesarean patients present with unexplained, worsening abdominal symptoms [16,17].

Clinicians must remain vigilant for atypical post-operative complications, particularly in global or resource-constrained settings. This case offers valuable clinical pearls: persistent abdominal pain, fever, or gastrointestinal distress in post-cesarean patients warrants a broad differential diagnosis, including non-gynecologic causes like PPU. Radiographic imaging should not be delayed when physical findings suggest peritonitis. Additionally, bedside ultrasound may be particularly useful in evaluating intra-abdominal pathology without subjecting the patient to radiation [18,19,20,21].

Several limitations should also be acknowledged. The patient’s prior medical, surgical, and pregnancy history was unknown due to her status as a migrant passing through Egypt, making it difficult to assess potential predisposing risk factors for ulcer formation. In addition, limited diagnostic resources influenced the availability of advanced imaging and laboratory investigations. Furthermore, the patient was lost to follow-up after discharge, limiting our ability to comment on long-term outcomes or perform additional etiologic evaluation such as testing for *Helicobacter pylori*.

In conclusion, this case contributes to the limited literature on PPU in the postpartum period and reinforces the importance of considering a wide differential diagnosis in post-operative patients. It highlights how rare but life-threatening conditions may mimic common post-operative complaints and underscores the value of prompt, resource-appropriate evaluation and intervention to prevent adverse outcomes.

This case report has several limitations. First, the patient’s detailed medical and ob-stetric history prior to presentation was unavailable, limiting assessment of predisposing factors such as prior *H. pylori* infection, NSAID use, or chronic gastrointestinal disease. Second, the diagnosis and management occurred in a resource-limited setting, which may have influenced the diagnostic timeline, access to imaging, and postoperative monitoring. Third, the absence of long-term follow-up data restricts evaluation of recovery quality and recurrence risk after surgical repair. Lastly, because this report presents a single case, the findings cannot be generalized but rather serve to highlight diagnostic and management considerations for similar clinical presentations.

## 4. Conclusions

Perforated peptic ulcer (PPU) in the post-cesarean period is an exceptionally rare but life-threatening complication that can mimic common postoperative conditions such as ileus or bowel injury. This case underscores the critical importance of maintaining a broad differential diagnosis when evaluating post-cesarean patients with persistent or worsening abdominal pain, fever, or gastrointestinal distress. Early imaging, particularly in the presence of peritoneal signs or hemodynamic instability, is essential to avoid delays in surgical intervention. In resource-limited settings, bedside ultrasound and plain radiographs can serve as valuable diagnostic tools. Prompt recognition and timely surgical management are key to improving outcomes. Ultimately, this case reinforces the need for vigilance among clinicians and the value of interdisciplinary collaboration in identifying atypical postoperative complications.

## Figures and Tables

**Table 1:** Timeline of the case development, presentation, management and recovery.

Timepoint	Clinical Events	Evaluation/Findings	Management
Day 0	Cesarean section performed with delivery of a healthy infant	No reported perioperative complications	Routine postoperative recovery
Postpartum Week 3 (Day ~21)	Patient developed abdominal pain, vomiting, and fever	Symptoms initially suspected to be gastroenteritis	Managed as outpatient with supportive therapy
Days 1–4 after symptom onset	Persistent abdominal pain and gastrointestinal symptoms	Progressive worsening despite outpatient treatment	No clinical improvement
Day 4–5 after symptom onset	Significant clinical deterioration	Hypotension (BP 80/40 mmHg), tachycardia (HR 140 bpm), fever (40 °C)	Hospital admission and resuscitation
Admission evaluation	Signs of systemic inflammatory response	WBC 36,000/µL; Hgb 10 g/dL; Platelets 101,000/µL; INR 1.84; Cr 1.4 mg/dL; Na 130 mmol/L	Broad-spectrum antibiotics and intravenous fluids initiated
Imaging	Suspicion for intra-abdominal pathology	Abdominal X-ray showing pneumoperitoneum, dilated bowel loops, and subcutaneous edema	Emergency surgical consultation
Emergency surgery	Exploratory laparotomy performed	3 cm perforated peptic ulcer identified	Peritoneal lavage and Graham omental patch repair
Postoperative Day 2	Clinical stabilization	Platelets improved to 122,000/µL; INR improved to 1.6	Continued ICU monitoring
Postoperative Day 3	Gradual clinical improvement	Patient tolerated oral fluids	Diet advanced
Postoperative Day 5	Continued recovery	Stable vital signs	Soft diet initiated
Postoperative Day 7	Recovery complete	Stable clinical condition	Discharged home

## Data Availability

The original contributions presented in this study are included in the article. Further inquiries can be directed to the corresponding author.

## Abbreviations

The following abbreviations are used in this manuscript:

COX	Cyclooxygenase
EGF	Epidermal Growth Factor
ICU	Intensive Care Unit
NSAID	Non-Steroidal Anti-Inflammatory Drug
PPU	Perforated Peptic Ulcer
PUD	Peptic Ulcer Disease
